# Evaluation and Comparison of Latent Health Risk Prediction Models for Clinical Triage: Protocol for a Mixed Methods Study

**DOI:** 10.2196/85437

**Published:** 2026-07-03

**Authors:** Morgan Roberts, Otso Pelkonen, Diana Shamsutdinova, Saskia C Sanderson, Pawel Renc, Hugh Logan Ellis

**Affiliations:** 1 Emergency Department, Bristol Royal Infirmary, Bristol, United Kingdom; 2 Department of Biostatistics and Health Informatics, Institute of Psychiatry, Psychology and Neuroscience, King's College London, IoPPN, 16 De Crespigny Park, London, SE5 8AB, United Kingdom, 44 (0)20 7848 000; 3 Department of Behavioural Science and Health, University College London, London, United Kingdom; 4 Department of Radiology, Massachusetts General Hospital, Boston, MA, United States; 5 AGH University of Krakow, Kraków, Poland; 6 Harvard Medical School, Boston, MA, United States

**Keywords:** artificial intelligence, clinical decision support systems, triage, machine learning, electronic health records, deep learning, frailty, clinical validation, mixed methods research, human-computer interaction

## Abstract

**Background:**

Clinical triage requires integrating multiple information sources to identify patients at risk of deterioration. Tools capturing global health assessments beyond disease-specific scores are being developed using either bottom-up aggregation of simple indicators or top-down machine learning from large datasets. Their alignment with expert clinical judgment remains poorly characterized.

**Objective:**

This study evaluates 2 latent health measurement approaches: Frailty Index-laboratory, a transparent bottom-up tool aggregating laboratory abnormalities via deficit accumulation theory, and ETHOS-ARES (Enhanced Transformer for Health Outcome Simulation-Adaptive Risk Estimation System), a transformer-based foundation model generating multidimensional patient representations from electronic health records. We assess whether each tool’s severity rankings align with clinical consensus and whether they offer utility in triage decisions.

**Methods:**

In this 3-phase mixed methods study, at least 30 clinicians across hospital specialties reviewed 20 emergency department presentations derived from Medical Information Mart for Intensive Care IV-Emergency Department. Phase 1 compared unaided clinician severity and urgency judgments against model outputs using Spearman rank correlation, with a Turing-inspired indistinguishability test assessing whether model rankings fell within the distribution of clinician assessments. Phase 2 allocated clinicians to receive Frailty Index-laboratory or ETHOS-ARES outputs, measuring anchoring effects via within-person pre-post comparisons and exploring clinical utility through semistructured interviews analyzed using the Framework Method.

**Results:**

Ethics approval was granted in June 2025 (KCL Research Ethics Office; MRSP-24/25‐48707). Recruitment began in October 2025 (32 clinicians recruited as of manuscript submission), with data collection expected to be completed in January 2026 and analysis planned for March or April 2026.

**Conclusions:**

This study will quantify model-clinician agreement, measure anchoring effects, and generate qualitative insights on utility, trust, and adoption. The findings will inform the implementation of latent health measurement tools in clinical practice and provide a framework for the early-stage evaluation of artificial intelligence–based clinical decision support systems.

## Introduction

### Background

Hospital clinicians working on call frequently face the challenge of deciding which patient to assess next from among multiple competing demands. A key component of this triage decision involves identifying the sickest patient: those most at risk of deterioration or in greatest need of urgent intervention. However, clinical urgency is not determined by illness severity alone. Clinicians must also weigh disease trajectories, available resources, the acuity of other patients, and the likelihood that early intervention will alter outcomes [1,2]. These multifaceted judgments, though central to clinical practice, are difficult to quantify and communicate.

To support such decisions, numerous tools have been developed to measure patient condition, ranging from organ-specific scores like the Glasgow Coma Scale for head injury [3] and the HEART score for chest pain [4] to more general assessments of physiological disturbance. The National Early Warning Score (NEWS) exemplifies the latter, using bedside vital signs to provide a standardized measure of acute illness severity that has been widely adopted in the United Kingdom and internationally [5]. While NEWS offers transparency and allows clinicians to factor in clinical context, it relies solely on vital signs and cannot access the broader information available in electronic health records (EHRs), where early markers of physiological disruption may be evident before vital signs deteriorate. Williams’s [5] 2022 review of NEWS anticipated the rise of more complex, data-driven scoring systems while emphasizing the need for such tools to inform clinical decisions rather than replace them.

With EHRs now ubiquitous in health care settings, there is growing interest in whether richer data sources can provide more comprehensive assessments of patient health. With the advent of machine learning, there has been much excitement surrounding the implementation of artificial intelligence (AI) in the diagnostic assessment of patients. A recent meta-analysis showed comparable performance between models and nonexpert clinicians; however, models were inferior to “expert” clinicians [6]. Despite this, overall patient health and illness severity represent a different task from diagnosis alone. Several tools designed for the prediction of risk, or “early warning,” have been developed in recent years and tested within the hospital settings, with promising results [7]. We sought to evaluate how clinicians perceive and use 2 contrasting approaches to this challenge. Wang et al [8] characterized 2 fundamental paradigms for leveraging such data: bottom-up methods that aggregate simple indicators across multiple domains to build composite pictures of health and top-down approaches that employ advanced computational techniques to learn complex health representations from large datasets. Either paradigm offers distinct advantages and limitations that may influence clinical utility and acceptance.

The Frailty Index-laboratory (FI-lab) exemplifies the bottom-up approach. First developed in 2014, grounded in Rockwood and Mitnitski deficit accumulation theory of frailty [9,10], the FI-lab calculates the proportion of available routine laboratory tests falling outside reference ranges at a given time point. This approach operationalizes the principle that health deficits accumulate with age and illness and that the proportion of deficits present predicts adverse outcomes [10,11]. A systematic review and meta-analysis by Sapp et al [11] demonstrated that FI-lab scores consistently predicted mortality, hospitalization, and other adverse outcomes across populations. More recently, Pasternak et al [12] have shown retrospective data demonstrating that increasing FI-lab scores have a strong association with both in-hospital mortality and mortality within the first year after hospitalization. A key strength is its relative transparency; simple inputs allow clinicians to pick apart how a value contributes to a score. This facilitates understanding and allows trust to be built. However, relying solely on laboratory data excludes a wealth of information available in clinical notes, imaging, and drug records.

ETHOS-ARES (Enhanced Transformer for Health Outcome Simulation-Adaptive Risk Estimation System) represents the top-down paradigm. This transformer-based foundation model, developed by Renc et al [13], tokenizes EHRs and learns multidimensional patient representations through self-supervised learning on large patient cohorts. It is able to capture complex nonlinear relationships between clinical data, identifying patterns that may not be self-evident through conventional aggregation. For clinicians, ETHOS-ARES can generate chronological timelines, identifying patterns across large patient cohorts to predict future outcomes and highlight risk states [13,14]. Despite being the more sophisticated model, ETHOS-ARES may present challenges of interpretation, with users unable to directly inspect the reasoning behind outputs. These 2 tools represent 2 contrasting approaches to the problem of latent health measurement.

Evaluating such tools presents distinct challenges. No single validated reference standard exists for “latent health status,” the underlying interplay of physiological reserve and illness burden that these tools attempt to measure. Traditional validation of tools against outcomes such as mortality or hospital admission is unable to capture the “in the moment” recognition of deterioration or urgency required for a real-time decision support tool [7,13,15]. This reflects a recognized “model-implementation gap.” Tools validated on retrospective data fail to translate into real-world clinical benefit because models that demonstrate good statistical performance fail to prove their clinical usefulness due to a failure to integrate into existing clinical workflows or gain clinician trust [16-18]. Successful clinical implementation requires not only predictive accuracy but also interpretability, transparency, and usability in an ethically robust manner. These requirements are highlighted by Tun et al [15] and recent consensus recommendations outlined by Labkoff et al [18]. Our previous work identified similar themes but additionally emphasized the ability to compare current health against a premorbid baseline [19]. Tools may well be validated in a retrospective setting or for general outcomes, but without real-time, clinician-centered testing, patients may not benefit. Our approach aims to address the implementation gap by evaluating clinician-model agreement in realistic triage scenarios.

This study, therefore, uses clinical triage—a scenario familiar to on-call clinicians—as a simulation to address 2 questions: (1) Do these tools’ assessments of patient health align with those of experienced clinicians, whom we treat as our reference standard in this context? and (2) Do clinicians find the tools useful when incorporated into triage decisions?

### Primary Objectives

Determine whether each tool’s severity scores and rankings align with clinician consensus on (1) resolved severity ranking and (2) clinical urgency (the order in which patients should be assessed). We hypothesize that both models will demonstrate a significant correlation with clinician consensus.

### Secondary Objectives

Assess whether tool exposure anchors clinicians toward tool outputs; test whether tool rankings are distinguishable from human rankings (Turing-inspired indistinguishability test); and explore, through qualitative interviews, how clinicians interpret and use the tools, their points of agreement or disagreement, and their perceptions of utility and limitations. We also hope to explore whether clinical consensus varies by clinical role (eg, surgeons vs physicians) and compare the 2 model outputs. However, this may be limited by recruitment and distribution of roles.

## Methods

### Key Terms

For the purposes of this study, we define the following terms. “Illness severity” refers to the current physiological burden placed upon a patient by their ongoing disease process at a given time point. “Clinical urgency” refers to the priority placed upon patient assessment or treatment, given the patient characteristics, disease trajectory, and the potential that early intervention will be of benefit. “Latent health status” refers to an underlying physiological reserve and cumulative health deficit burden that may not be directly observed but is indicated by multiple clinical factors. It represents not only baseline resilience to current or future physiological insults but also the accumulated clinical deficits that influence prognosis.

### Study Design

This is a 3-phase mixed methods study that employs a crossover mechanism to allow quantitative evaluation, in combination with a semistructured qualitative interview. It aims to use realistic clinical triage situations to test the real-time application of 2 computational tools measuring latent health status. Through this, we hope to explore how clinicians interpret and use tool output as part of their decision-making process.

### Participants

We will recruit at least 30 currently practicing clinicians to our study. This sample size was determined by both practical and analytical considerations. For the primary correlation analysis, power calculations indicate that with 20 patient cases, we have 80% power to detect a correlation of ρ=0.6 as statistically significant at α=.05. For the anchoring effect analysis (paired *t* test comparing exposed vs unexposed scores), a minimum sample of 30 clinicians (with 15 per arm) provides 80% power to detect a difference of approximately 2 rank positions between the groupings. The sample size also allows for limited subgroup analysis between types of clinicians (eg, surgeon vs physician).

We will aim to sample across roles: hospital physicians (internal medicine, emergency medicine), surgeons, across a range of grades of resident doctors (senior house officer to senior registrar), and consultants or attendings. Additional inclusion criteria are English-speaking individuals with no prior involvement in case selection or preparation, no prior experience of MIMIC dataset, and no involvement in the development of ETHOS or FI-lab. Recruitment will be conducted via hospital networks and professional contacts.

### Clinical Cases

#### Overview

Cases come from the MIMIC-IV-ED (Medical Information Mart for Intensive Care IV-Emergency Department) test split used during ETHOS-ARES development (held-out during training) [13,20]. MIMIC-IV-ED is a freely accessible, deidentified database of emergency department (ED) encounters from Beth Israel Deaconess Medical Center, a large academic medical center in Boston, Massachusetts. The database contains comprehensive clinical data including patient demographics, triage information, vital signs, laboratory results (including historical results prior to admission), imaging reports, and clinical notes collected during routine clinical care.

#### Case Selection Criteria

Cases were sampled at random from the MIMIC-IV-ED test split and assessed based on the following criteria: (1) adult patient aged 18 years or older, (2) admitted to the hospital via the ED, (3) available ED documentation, inpatient bloodwork, and scan results, and (4) presence of clinical time points that could reasonably occur as tasks or calls during an on-call shift (eg, new scan results available, clinical deterioration) until 20 appropriate cases had been found that were suitable for the study. Note that a minority of patients selected had no historical laboratory results.

#### Triage Time Point Definition

All clinical information presented to each assessor relates to a chosen “triage time point” in a patient’s hospital journey. Time points that represent a significant event within their clinical journey that would trigger a request for clinical assessment were selected; time points within intensive care were excluded. Time points were agreed upon by authors MR, HLE, and OP. Both FI-lab and ETHOS-ARES scores were calculated using only information available at the same time point.

#### Case Presentation Format

Materials are delivered as a slide deck rather than a full electronic patient record. Each case slide set includes an ED clerking; a structured history and a physical examination captured before ward arrival (sourced via the ED discharge summary but restricted to history and examination content only to avoid outcome leakage); laboratory results; and radiology reports, where applicable. Each opening slide contains a brief handover-style note highlighting pertinent aspects of medical history and investigation results. Inpatient progress notes and discharge outcomes are not shown. An example created without patient data is available in Multimedia Appendix 1.

### Tools Under Evaluation

#### Frailty Index-Laboratory

A non-AI algorithmic index based on deficit accumulation theory. It calculates the proportion of available routine laboratory tests outside the reference range aggregated at the time point [9,10]. For this study, 2 FI-lab plots were calculated: (1) a “chronic” plot based on available laboratory results up to 1 year before admission and (2) an “acute” plot based upon in-hospital blood tests available at the triage time point. For the majority of patients, this allows us to display an acute FI-lab score, relative to a “chronic” baseline, a functionality identified as desirable by our previous work [19].

#### FI-Lab Presentation to Participants

Participants will receive a short explanatory slide describing the theory behind its development, a summary of how it is calculated, and will be invited to see an FI-lab score for a patient they have previously assessed in the unexposed wing of the study as an example. During the task, the participant will be presented with a numerical score for each patient and their corresponding triage time point, a graph depicting the “severity/acute” FI-lab score over time leading up to the time point, and the FI-lab “chronic” score, if available, as a baseline on the graph.

#### Enhanced Transformer for Health Outcome Simulation-Adaptive Risk Estimation System

A transformer-based foundation model that learns multidimensional patient representations from EHRs. The model tokenizes clinical events and uses self-supervised learning to create embeddings that capture the complex relationships between clinical events [14]. The model can then be used to generate future timelines for each patient from any given time point as a multidimensional embedding vector that reflects the model’s expectation of the patient’s clinical course if the patient survives; this is used as a proxy for illness burden. This is expressed as a graph of “activity-based risk” over time up to the chosen triage time point. The reasoning behind the scores is not directly inspectable.

#### ETHOS-ARES Presentation to Participants

Participants will receive a short slide explaining the basic theory behind ETHOS-ARES training along with an explanation of how to interpret the graph. They will be invited to see an ETHOS score for a patient they have previously assessed in the unexposed wing as an example. During the task, the participant will be presented with a numerical score for each patient and their corresponding triage time point and a graph depicting the “severity/acute” ETHOS-ARES score over time leading up to the time point.

### Interview Allocation

### Overview

**Figure 1.**
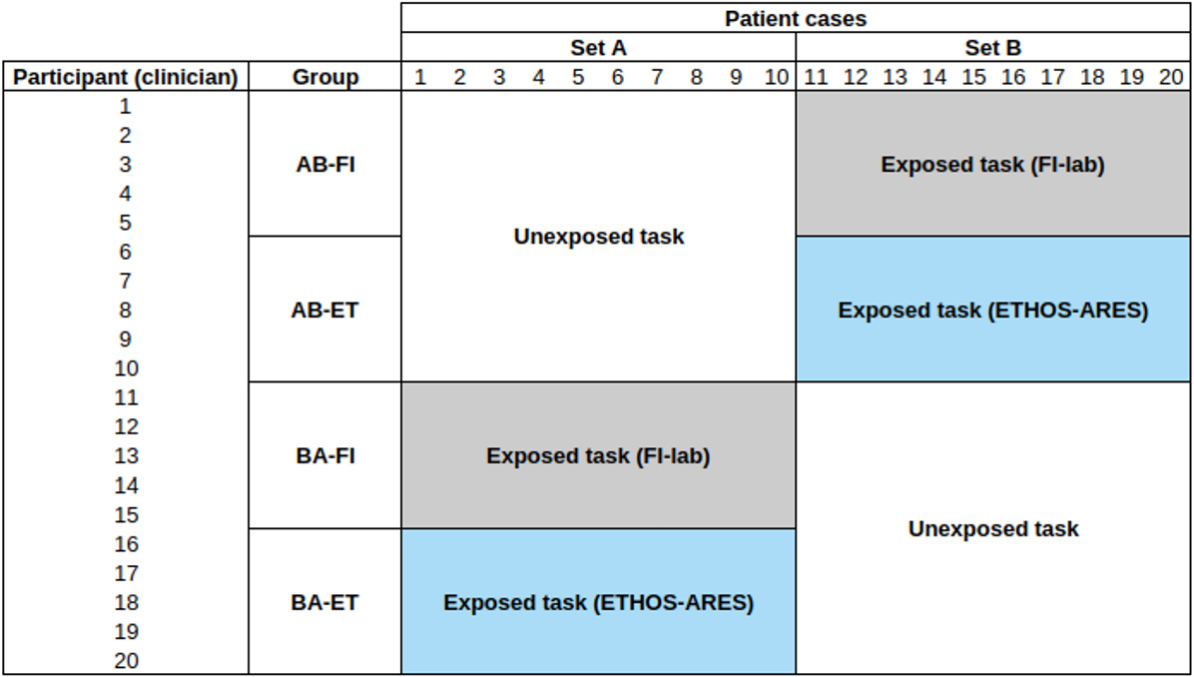
Example of interview allocation. For illustrative purposes, this example only shows 20 clinicians. Here, the first 10 clinicians are allocated into the unexposed scoring of the Set A cases and are exposed to 1 of the 2 models for Set B (AB arm). Similarly the latter 10 clinicians would first work through the Set B cases without model exposure before moving onto Set A with model exposure (BA arm). Unexposed scoring, shown on a white background, is conducted first. Exposed scoring is shown on a gray background for Frailty Index-laboratory (FI-lab) and blue background for ETHOS-ARES (Enhanced Transformer for Health Outcome Simulation-Adaptive Risk Estimation System).

Participants are allocated to 1 of 4 groups using sequential balanced allocation, with each interviewer assigning participants to groups in rotation to ensure approximately equal numbers across conditions (Figure 1). Groups are divided first by case sequence, for example, group AB is Set A (patients 1‐10) for unexposed followed by Set B (patients 11‐20) for exposed. The second grouping is according to which model the participant will be allowed to use for their assessment of patients, denoted by FI for FI-lab and ET for ETHOS-ARES. Thus, we will have 4 groups for participants to be allocated to: (1) AB-FI, (2) AB-ET, (3) BA-FI, and (4) BA-ET.

#### Phase 1: No Tool Exposure (Unexposed)

The interview is divided into 3 phases. For an overview, see Textbox 1. In phase 1, we propose model evaluation by comparison against the assessments of working clinicians. These clinicians will be given the information for 10 patients to consider in the context of a realistic clinical triage scenario. During the first task, they will be asked to score patients using a modified form of the American Society of Anesthesiologists physical status classification. Here, we will introduce a decimalized version of this well-known score to allow clinicians to more accurately make their assessment of a patient’s health status. This will be compared directly with the model outputs.

The second task of phase 1 will require our clinicians to rank the same patients in order of clinical urgency, effectively, listing them in order from “most urgently in need of care” to “safe to wait.” We will compare this ranking to that of the previous task, the average highest perceived severity to lowest, and the ranked scores from our models. This task more accurately reflects the work of a clinician, and through it we hope to see if our models differ from our clinician standard.

Textbox 1. **Overview of interview structure and methodology.**

**Introduction**

- Participating clinicians are given a series of introductory slides that explain the study and the models under evaluation. They will be familiarized with the “handover” scenario.

**Allocation**

- Clinicians are allocated to 1 of 4 groupings that determine the order in which they will see patient groups and model outputs.

**Phase 1**

- Task 1a: Overall severity score (1-5) per case (American Society of Anesthesiologists-style anchors provided).
- Task 1b': Resolved severity ranking of the same 10 cases (participants break ties to produce a strict 1-10 order).
- Task 2: Clinical urgency ranking (1=see first … 10=see last).

**Phase 2**

- Short slide-deck explainer on the assigned tool.
- Display tool outputs (Frailty Index-laboratory score or Enhanced Transformer for Health Outcome Simulation-Adaptive Risk Estimation System severity score) alongside each case.
- Repeat Task 1a, Task 1b', and Task 2, allowing model outputs to be incorporated into clinical reasoning.

**Phase 3**

- Brief view of the other tool’s outputs for 2 to 3 exemplar cases; short survey comparing perceived utility.
- Semistructured interview (15-25 min) covering perceptions of the tool, what was good, whether participants trusted it, and what was bad.

#### Phase 2: Tool Exposure and Integration (Exposed)

For phase 2 of our study, we seek to learn how clinicians interpret model output and incorporate it into their decision-making. Clinicians will receive information for a further 10 patients; the tasks will be identical; however, in this instance, the clinicians will be introduced to 1 of the 2 models before being shown model outputs for each patient. Clinicians will be allocated into receiving either the FI-lab output or the ETHOS-ARES output for their second 10 patients.

#### Phase 3: Cross-Exposure and Interviews

After clinicians have submitted their scores and rankings for the second 10 patients, they will be shown the alternate model output for comparison alongside both model outputs for the first 10 patients for their own review. Following this, we will follow a semistructured interview to gather qualitative data about the clinicians’ perceptions regarding the utility, benefits, and perceived risks of the tool in question.

### Quantitative Methods

Phases 1 and 2 of our study yield quantitative data, as illustrated in Figure 1, showing the participant groups. A corresponding dataset was collected for each participant’s severity scores, severity rankings, and clinical urgency rankings. For example, a clinician allocated to the AB-FI group would score Set A cases unexposed (without seeing the models’ scores) and Set B while seeing the output of the FI-lab algorithm.

### Primary and Secondary Outcomes

For tabular summaries of primary and secondary outcomes and their corresponding statistical methodologies, see Tables1  2, respectively.

**Table 1.** Summary of primary outcomes and statistical methodologies.

Primary outcome	Corresponding hypothesis	Methodology
1A: Agreement between tool-generated rankings and polled clinician consensus for clinical urgency (the order in which the patients should be seen)	Hypothesis 1A: There is no significant correlation between the models’ clinical urgency rankings and the clinicians’ consensus	Spearman correlation for both ETHOS-ARES^a^ vs consensus clinical urgency and FI-lab^b^ vs consensus clinical urgency
1B: Agreement between tool-generated rankings and polled clinician consensus for resolved severity ranking	Hypothesis 1B: There is no significant correlation between the models’ resolved severity rankings and the clinicians’ consensus	Spearman correlation for both ETHOS-ARES vs consensus severity ranking and FI-lab vs consensus severity ranking

a ETHOS-ARES: Enhanced Transformer for Health Outcome Simulation-Adaptive Risk Estimation System.

b FI-lab: Frailty Index-laboratory.

**Table 2.** Summary of secondary outcomes and statistical methodologies.

Secondary outcome	Corresponding hypothesis	Methodology
2A: Clinical Turing test, that is, the degree to which model output is distinguishable from clinicians’ assessment	Hypothesis 2A: Model rankings are not the outliers in the observed distribution of the clinicians’ ratings	*z* score analysis: compute distance of model and participant rankings from unexposed consensus. Permutation test with logistic regression to test if a classifier can distinguish “clinician” vs “model” label using z scores. Model is distinguishable if *p*_model_>*p*_*i*_ for >90% of participants.
3A: Anchoring effect of model on consensus level	Hypothesis 3A: Correlation of the consensus rankings with the model rankings is stronger for the exposed rankings than for unexposed	Compute absolute differences between model scores and both unexposed and exposed consensus rankings; 1-sided paired *t* test to test if exposed consensus is closer to model than unexposed consensus
3B: Anchoring effect of model on individual level	Hypothesis 3B: Participant-level correlation with the model rankings is stronger for the exposed rankings than for unexposed	Calculate each participant’s correlation with model scores when unexposed versus exposed; paired 1-sided *t* test to test if mean correlation increases in the exposed scenario (*P*<.05 indicates significant anchoring)
4A: Correlation between models	Hypothesis 4A: There is a significant correlation between the models’ rankings	Spearman correlation test between the ETHOS-ARES^a^ and FI-lab^b^ scores for the 20 cases
5A: Correlation between clinicians’ assessment of severity and clinical urgency	Hypothesis 5A: There is a significant correlation between clinicians’ assessment of a patient’s severity and clinical urgency	Spearman correlation test between the consensus severity and consensus urgency scores; both consensus scores will be computed using unexposed scores

a ETHOS-ARES: Enhanced Transformer for Health Outcome Simulation-Adaptive Risk Estimation System.

b FI-lab: Frailty Index-laboratory.

#### Primary Outcomes

Agreement between tool-generated rankings and polled clinician consensus for the following:

1A: Clinical urgency ranking (the order in which the patients should be seen)
1B: Resolved severity ranking (derived from severity scores with ties broken by the clinician)

#### Statistical Analyses

##### Main Hypothesis

Model scores are similar to the clinicians’ consensus. In particular, there is a statistically significant correlation between the clinicians’ consensus (mean scores) and the model scores.

##### Method

We will compute the pooled clinical consensus by averaging raw unexposed severity scores provided by the clinicians allocated to the AB arm for Set A and across the BA arm for Set B. These averages will be used to create a consensus ranking of severity. A Spearman correlation test will be used to estimate the correlation between the consensus and the ETHOS and FI-lab model rankings with corresponding CIs, where subsequently a *P* value <.05 would indicate that we reject the hypothesis of no correlation between the consensus and model scores. Similarly, we will conduct a correlation analysis for clinical urgency by comparing the model-provided rankings to the clinician-provided consensus clinical urgency rankings.

In total, this will result in 4 correlation tests: ETHOS vs consensus clinical urgency, ETHOS vs consensus severity ranking, FI-lab vs consensus clinical urgency, and FI-lab vs consensus severity ranking.

##### Power Calculations

Power calculations conducted using the “pwr” and “pwrss” R packages [21,22] indicate that assessing 20 patients gives 80% power to detect correlations >0.6 for Spearman correlation. This power is reasonable, given that (1) the pilot scores indicate correlations ranging from 0.62 to 0.90 (varying between the models and severity and urgency marks) and (2) a higher bar for the detectable correlation will ensure a strong indication that the FI-lab or ETHOS scorings align with the practitioners’ consensus.

### Secondary Outcomes

Below are outlined the hypotheses and proposed statistical methods for each of our secondary outcomes.

1. Turing test: model rankings are not distinguishable from clinicians’ rankings
  - Hypothesis 1. Model rankings are not the outliers in the observed distribution of the clinician ratings.
  - Method: For each participant and for the model, we compute *z* scores of how far their estimates are from the participant’s unexposed consensus (excluding the model), expressed in SDs. We will then visually inspect the model and participants’ *z* scores to investigate whether the models’ *z* scores are the outliers.
  - Permutation test with a classifier regression (eg, logistic regression). Here, we test whether a classifier regression can predict the label “clinician” or “model” using *z* scores as predictor. First, a logistic regression is fitted, and we compute an estimation for the probability that the model’s scores belong to the label “model,” *p*_model_. As we only have 1 vector of scores for the model, we cannot perform a standard train or test evaluation and instead utilize a permutation test. In this test, we will choose a random clinician to become a “model” and compute their separability from the rest of the dataset in a similar way, yielding comparator statistics *p_i_* for all participating clinicians. We will conclude that the model is statistically distinguishable from the participants if *p*_model_ > *p_i_* for more than 90% of the participants.
2. Anchoring effect of the models
  - Hypothesis 2A (group-level impact): Exposing participants to the model rankings makes their consensus closer to the model’s scores. That is, the mean absolute difference between the consensus and the model scores is lower for the exposed consensus than for the unexposed.
  - Method: As in the test for the main hypothesis, we first compute the pooled consensus rank for each of the cases, averaging unexposed rankings across the participants, for example, (*u*_1_,…, *u*_20_), for each of the rated cases. Similarly, the exposed consensus is computed (*e*_1_,…, *e*_20_). The absolute differences between the model scores (*m*_1_,…, *m*_20_) and the exposed and unexposed consensus will represent the closeness to the model in these scenarios, *d*_unexposed_ = (abs (*u*_1_ − *m*_1_),…, abs (*u*_20_ − *m*_20_)), *d*_exposed_ = (abs (*e*_1_ − *m*_1_),…, abs (*e*_20_ − *m*_20_)).
  - Finally, we perform a 1-sided paired *t* test for *d*_exposed_ and *d*_unexposed_ to investigate whether the difference is smaller for the exposed consensus than for the unexposed, Mean (*d*_exposed_) *<* Mean (*d*_unexposed_).
  - Power calculations show that with 30 clinicians (15 per arm) and 20 cases, we will be able to detect (a rather large) difference of over 2 rank points with 80% power. That is, if this test does not reach statistical significance, it could be due to insufficient power to detect a smaller impact of the models on consensus.
  - Hypothesis 2B (individual-level impact): The clinician-level correlation with the model rankings is stronger for the exposed rankings than for the unexposed.
  - Method: For each participant, we first compute the correlation of their ranks with the model scores. Participants in the AB arm will have an “unexposed” correlation with model scores for Set A and an “exposed” correlation with Set B, and vice versa for BA; therefore, each participant will have 1 “unexposed” and 1 “exposed” correlation. Second, we perform a paired 1-sided *t* test to investigate if the mean correlation has become higher in the exposed scenario. *P* value <.05 will imply a statistically significant difference.
3. Correlation between the models
  - Hypothesis 3: There is a significant correlation between the models’ rankings.
  - Method: Spearman correlation test between the ETHOS-ARES and FI-lab scoring for the 20 cases. *P* value <.05 will indicate that we can reject the hypothesis that the correlation is insignificant (zero). Similar power calculations as for the main hypothesis imply that assessing 20 patients gives 80% power to detect correlations greater than >0.6 as statistically significant.
4. Correlation between illness severity and clinical urgency as rated by clinicians
  - Hypothesis 4: There is a statistically significant correlation between the clinicians’ consensus for severity and urgency.
  - Method: Spearman correlation test between the consensus severity and consensus urgency scores. Both consensus scores will be computed using the unexposed results. Similar power calculations as for the main hypothesis imply that assessing 20 patients gives 80% power to detect correlations >0.6 as statistically significant.

### Qualitative Methods

Interviews will be audio-recorded. The audio recordings will be transcribed verbatim. The transcripts will be analyzed by the research team using thematic analysis. Published guidance, such as Braun and Clarke steps to thematic analysis [23,24], will be followed when analyzing the data to maintain scientific rigor. NVivo [25] will be used to manage the data and facilitate coding.

We will use the Framework Method. A short a priori codebook will be drafted from the aims (integration of tool outputs; agreement/disagreement reasons; perceived anchoring; confidence/trust; workflow impact) and refined on pilot transcripts. Two researchers will (1) independently review all transcripts, (2) joint-code approximately 20% to calibrate the codebook and resolve differences by discussion, and then (3) code the remaining data independently. Data will be charted into a matrix (cases × codes) to compare across clinicians and cases. We will present exemplar quotations, conduct deviant-case analysis, and maintain an audit trail (codebook changes and decisions). We will create joint displays aligning quantitative disagreement with qualitative explanations. Reporting will follow COREQ (Consolidated Criteria for Reporting Qualitative Research) [26].

### Ethical Considerations

Ethics approval was granted by the King’s College London Research Ethics Office, Minimal Risk Registration MRSP-24/25‐48707. Written informed consent will be obtained from clinician participants; case materials in MIMIC have already been deidentified; no patient-identifiable data will be shown. No financial compensation will be provided to participating clinicians. Deidentified case materials will be held in secure, access-controlled storage. Audio recordings will be transcribed, identifiers removed, and the transcripts stored securely.

## Results

Study materials, including clinical case presentations derived from MIMIC-IV-ED and semistructured interview guides (Multimedia Appendix 2), were developed between April and September 2025.

Recruitment of clinician participants commenced in October 2025 via hospital networks and professional contacts. Data collection began in October 2025 and was completed in January 2026, with 32 clinicians recruited across medical, surgical, and critical care specialties. Quantitative and qualitative analyses are planned for February to March 2026, with results anticipated for publication in mid-2026.

## Discussion

### Anticipated Findings

We anticipate that both FI-lab and ETHOS-ARES severity assessments will demonstrate a statistically significant correlation with clinician consensus, though the degree of agreement may differ between the 2 tools. FI-lab’s transparency and grounding in deficit accumulation theory may facilitate closer alignment with clinician reasoning for patients whose illness severity is predominantly reflected in laboratory derangements. ETHOS-ARES, by contrast, may capture subtler patterns across the clinical record that clinicians recognize but struggle to articulate. The qualitative component will provide insight into how clinicians interpret and integrate these different forms of computational health assessment into their existing decision-making frameworks.

### Comparison to Prior Work

This study builds on a growing body of work addressing the model-implementation gap in clinical AI [16-18]. While previous studies have validated clinical prediction tools against outcomes such as mortality and hospital admission, few have examined whether tool outputs align with the real-time clinical judgments that inform triage decisions. Our approach differs from conventional validation by treating clinician consensus as the reference standard rather than retrospective outcomes, reflecting the practical reality that these tools must be interpretable and trustworthy to the clinicians who will use them. The Turing-inspired indistinguishability test represents a novel methodological contribution, assessing not merely whether tool outputs correlate with clinical judgment but whether they are functionally indistinguishable from it. Recent consensus recommendations have emphasized the importance of clinician-centered evaluation prior to prospective deployment [19]; this protocol operationalizes that principle.

### Strengths and Limitations

Key strengths of this study include the mixed methods design, which enables both quantitative evaluation of tool performance and qualitative exploration of clinical reasoning. The use of realistic triage scenarios with standardized case materials provides ecological validity while controlling for information asymmetry between participants. The crossover design allows within-participant comparison of anchoring effects, and the inclusion of clinicians across medical, surgical, and critical care roles supports examination of whether professional background influences tool interpretation.

Limitations include the use of simulated rather than real-time clinical scenarios, which may not fully capture the time pressure and cognitive load of actual on-call decision-making. The sample size, while adequate for the primary correlation analysis, limits power for subgroup analyses between clinical roles. Cases are drawn from a single United States academic center (MIMIC-IV-ED), which may limit generalizability to other health care settings and populations. Presenting cases as slide decks rather than within a live EHR environment may influence how clinicians engage with the clinical information. Finally, clinicians will only have a limited amount of time to familiarize themselves with model outputs; however, a more extended training time would affect recruitment.

### Future Directions

Findings from this study will inform the design of prospective validation studies testing these tools in real-time clinical environments. The qualitative data will guide interface design and implementation strategies for clinical decision support tools based on latent health measurement. Permitting sufficient sample size, subgroup analyses may reveal whether different clinical roles demonstrate distinct patterns of agreement with model outputs or differential susceptibility to anchoring effects.

### Conclusions

This mixed methods protocol provides a framework for the early-stage clinical evaluation of latent health measurement tools prior to prospective deployment. By evaluating both a transparent, algorithmic approach (FI-lab) and an opaque, machine learning–based approach (ETHOS-ARES) within the same clinical scenarios, the study addresses fundamental questions about how different computational paradigms align with clinical reasoning and whether model exposure influences subsequent clinical decision-making.

## Supplementary material

10.2196/85437 Multimedia Appendix 1 Synthetic case: an example of a patient used in the interview and the associated FI-labs and ETHOS ARES outputs. This case is entirely synthetic, and no patient data has been used in its creation.

10.2196/85437 Multimedia Appendix 2 Guide used by interviewers.

## Abbreviations

AI: artificial intelligence
COREQ: Consolidated Criteria for Reporting Qualitative Research
ED: emergency department
EHR: electronic health record
ETHOS-ARES: Enhanced Transformer for Health Outcome Simulation-Adaptive Risk Estimation System
FI-lab: Frailty Index-laboratory
MIMIC-IV-ED: Medical Information Mart for Intensive Care IV – Emergency Department
NEWS: National Early Warning Score
