# Supplementary material for: Evaluation and Comparison of Latent Health Risk Prediction Models for Clinical Triage: Protocol for a Mixed Methods Study
Source: JMIR Res Protoc. 2026 Jul 3;15:e85437. doi: 10.2196/85437 (PMC13331393; doi:10.2196/85437)
Supplement: Multimedia Appendix 1 [file resprot-v15-e85437-s001.pdf]

Patient 1
Maya Ellery
61847205

29F. Severe hypertension, advanced CKD, inflammatory disease flare, worsening acidosis

All names, identifiers, trajectories and investigations in this deck are synthetic and for illustrative publication use only.

FI-lab:

Chronic: 0.36

Acute : 0.58

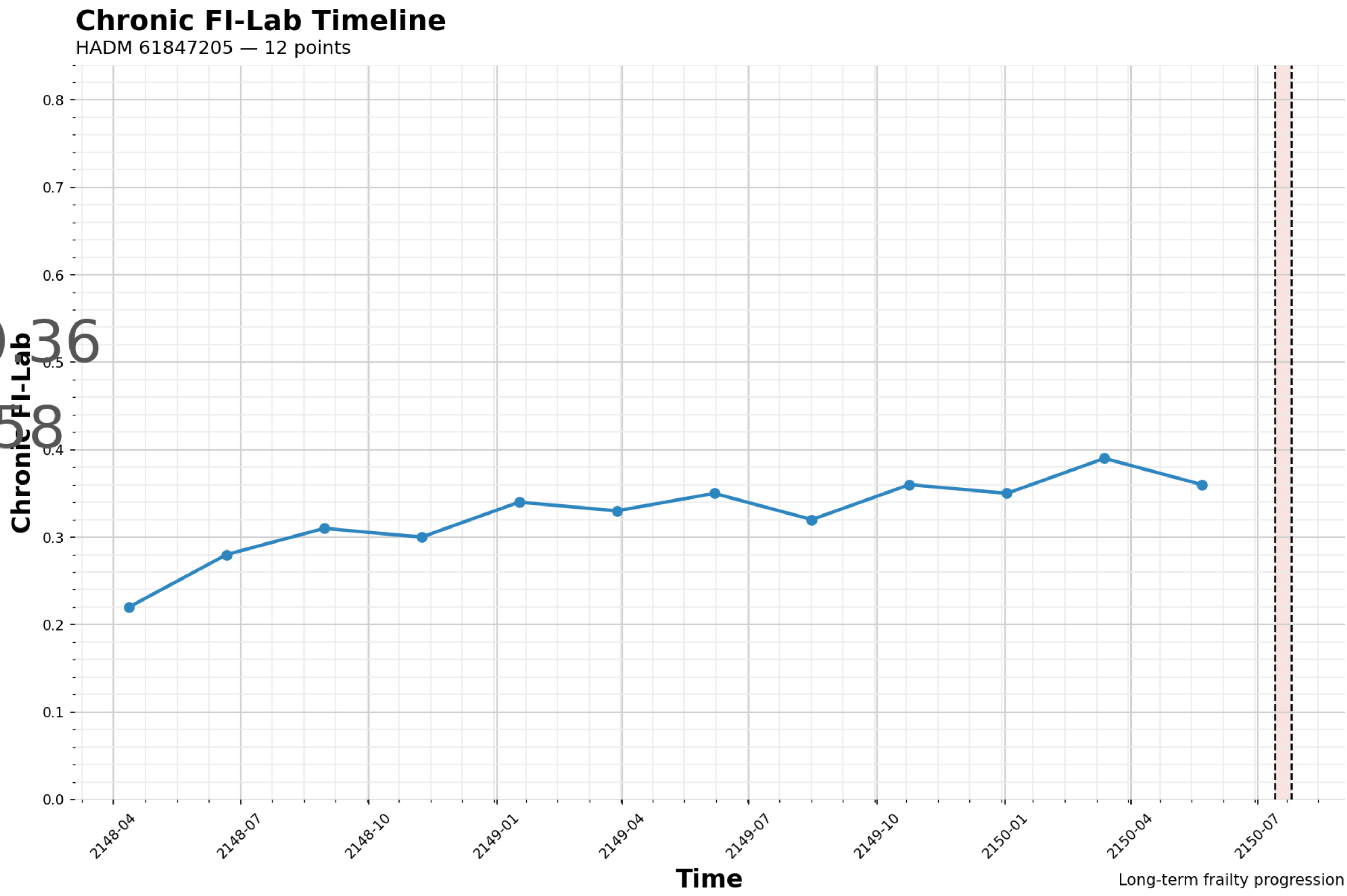

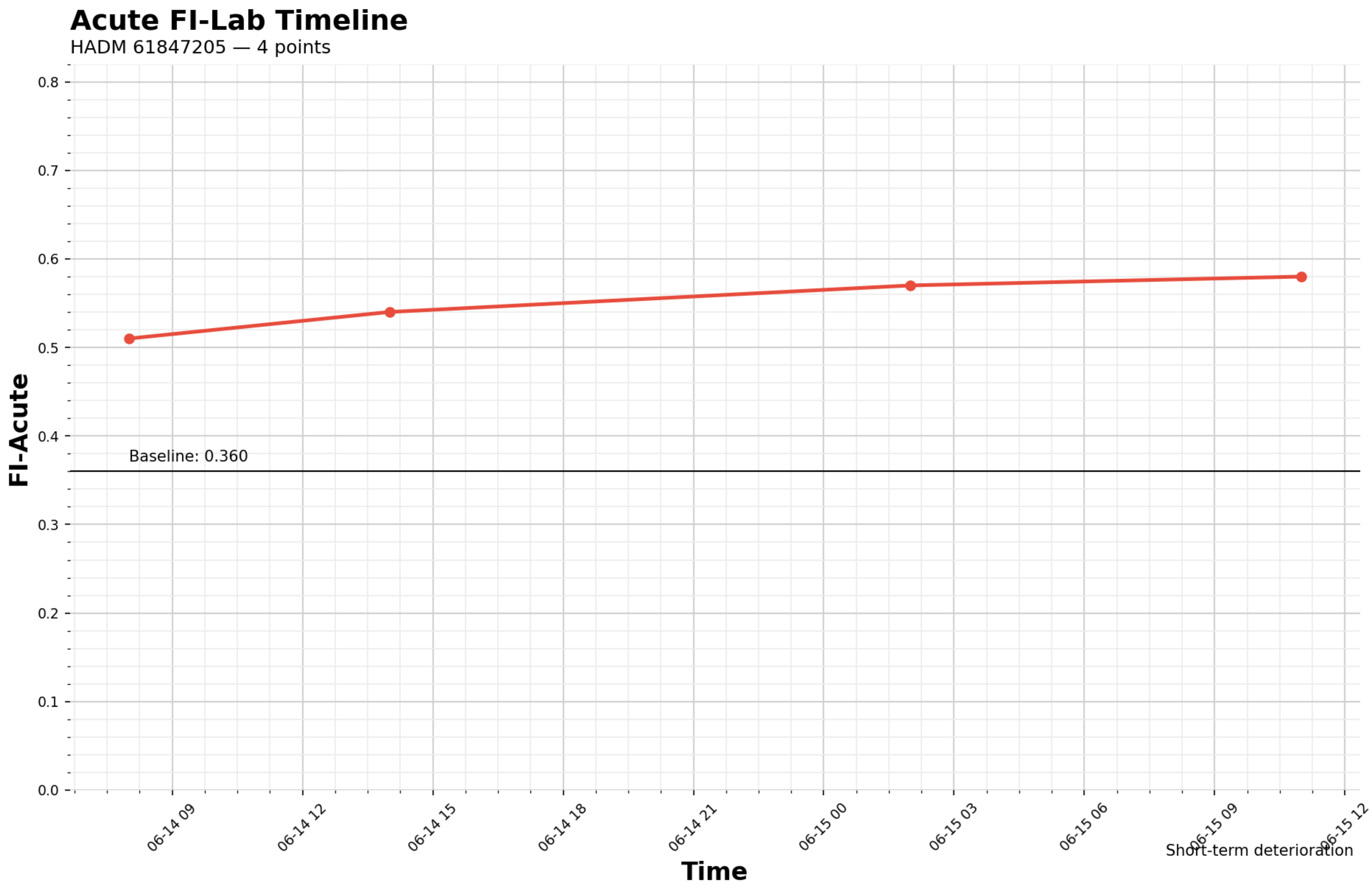

Acute FI-Lab Timeline
HADM 61847205 — 4 points
FI-Acute
0.0
0.1
0.2
0.3
0.4
0.5
0.6
0.7
0.8
Baseline: 0.360
06-14 09
06-14 12
06-14 15
06-14 18
06-14 21
06-15 00
06-15 03
06-15 06
06-15 09
06-15 12
Short-term deterioration
Time

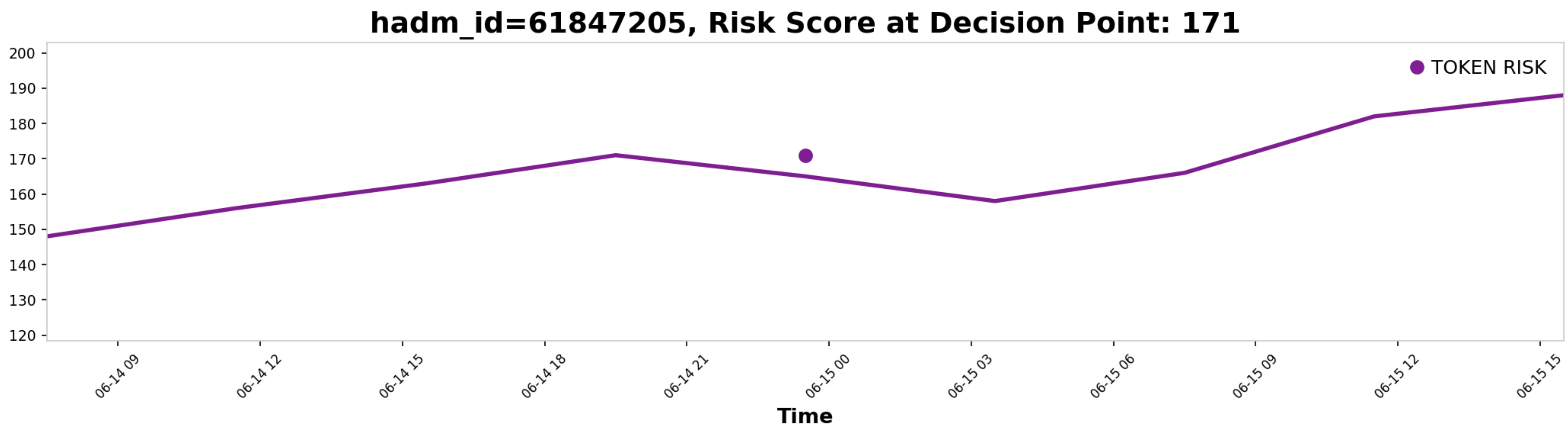

https://example.org/synthetic/case-a-61847205

ED Hx

History of Present Illness:

29 year old woman with mixed connective tissue disease and advanced chronic kidney disease presented with escalating right groin pain, nausea, reduced oral intake and severe hypertension. She had been discharged five days earlier after treatment for fluid overload and worsening renal function. Since leaving hospital she had struggled to tolerate her usual antihypertensive regimen because of vomiting and poor appetite.

On arrival she was hypertensive to 228/136 with preserved oxygen saturation and no focal neurological deficit. Venous blood gas showed a pH of 7.24 with bicarbonate 15. Potassium was 5.6 and creatinine 742 micromol/L. ECG showed left ventricular hypertrophy without new hyperkalaemic changes. Analgesia improved the groin pain, but biochemical derangement persisted despite fluids and medical treatment. Nephrology reviewed her in the ED because urgent dialysis was being considered if acidosis or potassium worsened.

The right groin pain had been investigated previously with negative Doppler ultrasound and plain films. There were no paraesthesiae, no weakness and no bowel or bladder red flags. She denied chest pain, headache or visual disturbance.

Synthetic illustrative case for publication use

PMHx

- Mixed connective tissue disease with previous lupus nephritis and intermittent serositis
- CKD stage 5 with progressive decline over two years. Dialysis access planning completed but renal replacement not yet started
- Severe hypertension with one previous PRES-like admission
- Chronic anaemia of renal disease
- Previous catheter-associated upper extremity thrombosis
- Secondary hyperparathyroidism and renal bone disease

DHx

- Amlodipine 10 mg orally once daily
- Labetalol 400 mg orally three times daily
- Hydralazine 50 mg orally three times daily
- Prednisolone 5 mg orally once daily
- Mycophenolate mofetil 500 mg twice daily
- Sodium bicarbonate 1 g orally three times daily
- Sevelamer 800 mg three times daily with meals
- Calcitriol 0.25 microgram once daily
- Oxycodone orally as required for pain

O/E

Vital Signs

- Temp 36.9°C, HR 88, BP 182/108 after treatment, RR 18, SpO_2 99% on room air

General Inspection

- Alert, fatigued, mildly nauseated, no respiratory distress

HEENT

- Moist mucous membranes
- No papilloedema or meningism
- Mild periorbital puffiness

Cardiovascular

- Regular rhythm
- Soft ejection systolic murmur
- No peripheral oedema

Respiratory

- Chest clear bilaterally with good air entry

Abdomen

- Soft, non-tender, no guarding
- No ascites
- Bowel sounds present

Musculoskeletal

- Right groin pain on resisted hip flexion but preserved passive range of movement
- No erythema or swelling of the joint

Neurological

- Orientated and conversational
- No focal weakness
- No sensory level or saddle anaesthesia

Skin

- Scattered post-inflammatory hyperpigmentation over forearms, no active rash

Bloods

Bloods

Test	Value	Reference
Sodium	136	135-145
Potassium	**5.6**	3.5-5.1
Bicarbonate	**15**	22-29
Urea	**24.8**	2.5-7.8
Creatinine	**742**	45-90
Calcium	**2.05**	2.15-2.55
Phosphate	**2.1**	0.8-1.5
Haemoglobin	**86**	115-160
Platelets	**141**	150-400
WBC	7.9	4.0-11.0
INR	**1.3**	0.9-1.2
pH	**7.24**	7.35-7.45

Synthetic illustrative case for publication use

Historical Lab Results

Historical Lab Results

Lab	2148-11-02	2149-03-18	2149-07-27	2149-10-11	2150-01-09	2150-05-15
Bicarbonate	19	18	17	16	16	15
Potassium	5.0	5.1	5.3	5.2	5.4	5.6
Creatinine	610	644	681	705	731	742
Urea	18.2	19.4	20.5	22.2	23.5	24.8
Haemoglobin	93	92	90	88	87	86
Platelet Count	176	170	161	154	148	141
Albumin	33	32	31	31	30	29
Urine Protein	2+	2+	3+	3+	3+	3+

Synthetic illustrative case for publication use

1.1. MRI pelvis and right hip

INDICATION: Persistent atraumatic right groin pain in a patient with advanced renal disease and inflammatory connective tissue disease.

TECHNIQUE: Multiplanar MRI pelvis and right hip without contrast.

FINDINGS:
There is no acute fracture, no femoral neck stress injury and no evidence of avascular necrosis. Trace right hip joint fluid is present without synovial thickening. Mild oedema is seen within the adductor origin and iliopsoas tendon insertion, in keeping with low grade strain. Marrow signal is diffusely heterogeneous but symmetrical, compatible with renal osteodystrophy. No drainable collection, no aggressive osseous lesion and no sacroiliitis.

IMPRESSION:
1. No acute bony abnormality of the right hip.
2. Mild adductor and iliopsoas insertional oedema, likely mechanical.
3. Background marrow changes consistent with chronic renal osteodystrophy.
