# Supplementary material for: Evaluation and Comparison of Latent Health Risk Prediction Models for Clinical Triage: Protocol for a Mixed Methods Study
Source: JMIR Res Protoc. 2026 Jul 3;15:e85437. doi: 10.2196/85437 (PMC13331393; doi:10.2196/85437)
Supplement: Multimedia Appendix 2 [file resprot-v15-e85437-s002.pdf]

Participant task

On-call simulation and interview

Contents

- Materials: consent form and data collection links
- Introduction: scenario and task outline
- Pre-task questions
- Task A
- Task B

Materials

Consent form: https://forms.gle/R4Gg8uxqYsHE4seVA

Data collection: https://ovjpel.github.io/research-resources/fi-lab/patient-task.html

- Prepopulate by adding e.g. "?participantId=001&participantGroup=1"
- participantGroup 1 for AB-FI (2 for AB-ET, 3 for BA-FI, 4 for BA-ET)
- Participant can fill manually as back-up option

Introduction: Scenario

It is a Saturday. You are a resident doctor working in a UK district general hospital. You are part of the medical on-call team, alongside the on-call registrar. Unfortunately, everyone else in the medical on-call team has called in sick.

Your registrar suddenly tells you she forgot she had a wedding to attend to - her own. She leaves you with a list of patients awaiting their weekend review and apologises for the lack of any formal handover, instead pointing out the brief summaries she wrote on her own list. You barely have time to congratulate her on her big day before she is out of the door. The site manager is breathing down your neck; you'll have to be efficient in your work.

Introduction: Task outline

- During this task, you will evaluate a total of 20 patients in terms of their unwellness as well as clinical urgency
  - The patients are split into two groups of 10 each, A and B
- You may be asked questions about the patients and your evaluation process
- You will be provided the information about each patient via a slide deck resembling a typical EHR
- The task and interview is recorded (audio only)
- Please ask your interviewer if you have any questions, although note that some task-related information will only become available as you progress

Pre-task questions

How many years of out of medical school are you? Are you in specialty training or a consultant role in a specialty; which specialty?

What EPR systems are you familiar with? Describe the systems you've worked with the most (whether paper, partly digital, fully digital).

Task A: First set of 10 patients

- Review the patients’ notes
- Assign each patient a number between 1.0 and 5.0 (decimals included; avoid ties) corresponding to how unwell or “sick” you consider them to be, using the following ASA-inspired scale:
    1. Healthy
    2. Mild systemic insult
    3. Severe systemic insult but stable
    4. Unstable patient
    5. Moribund without immediate intervention; peri-arrest
- Rank the patients in the order you would review them
- Maximum allocated time: 20 minutes

Waiting slide

Move to next slide once the task has been completed to the participant’s satisfaction or maximum allocated time has passed

Task A: Questions to participant

1. How did you find the process of assessing and prioritising the patients?
2. How difficult or complex would you say the cases were?

Task B: Introduction

- The task for the remaining patients is identical to the first, with one difference:
- Alongside the rest of the clinical information, you will be provided the output of an algorithm called FI-lab
- The following slide outlines the algorithm in order to explain the metric you are provided with

Task B: Introduction

- The task for the remaining patients is identical to the first, with one difference:
- Alongside the rest of the clinical information, you will be provided the output of an algorithm called ETHOS-ARES
- The following slide outlines the algorithm in order to explain the metric you are provided with

Task B: Introduction

- The task for the remaining patients is identical to the first, with one difference:
- Alongside the rest of the clinical information, you will be provided the output of an algorithm
- The following slide outlines the algorithm in order to explain the metric you are provided with

FI-lab

- FI-lab is an measure of a patient's status based on their laboratory test results: it corresponds to the portion of laboratory tests whose results do not lie in the normal range
- Recency of each lab test is considered with respect to how long it is physiologically valid for - for example, an old HbA1c will remain relevant for an FI-lab score for longer than a neutrophil count
- To illustrate, for a patient whose FI-lab score is 0.2, 20% of the considered lab tests are *abnormal* or *outside of the reference range* (higher score = higher proportion of derangement)

ETHOS-ARES: Basic information

- ETHOS-ARES is an AI algorithm similar in principle to large language models like ChatGPT
- However, instead of being trained on large amounts of text, ETHOS-ARES has been trained on large numbers of patient timelines
- Instead of generating words to create paragraphs, ETHOS-ARES generates clinical events to create patient timelines
- This allows ETHOS-ARES to generate countless “hypothetical future timelines” for a given patient; these hypothetical timelines and their statistics allows for derivation of clinically relevant measures

ETHOS-ARES: How to read the plot

Read the plot as a timeline of “activity-based risk” leading up to the decision point, which is the right-most dot.

The vertical scale is a proxy for clinical concern derived from ETHOS: it reflects the expected number of upcoming clinical events if the patient survives. Higher values generally mean more anticipated tests/procedures and therefore greater illness burden.

Emphasis should be on the trend and magnitude of changes rather than any single value: rises suggest escalating concern, falls suggest potential stabilization. Scrutinize sudden spikes or drops, which usually indicate a new result, order, or intervention worth reviewing at those timestamps. Use the trajectory to focus chart review and to interpret the current point in context, keeping in mind this is a proxy and should be combined with clinical judgment.

ETHOS-ARES: Optional supplementary information

- ETHOS-ARES is a transformer architecture very similar to GPT-2 (size ~124M parameters)
- It was trained on the MIMIC dataset (>400,000 hospitalisations, >200,000 patients) after the dataset was processed into tokenised, chronological patient timelines

Task B: Second set of 10 patients

- Review the patients’ notes and FI-lab assessments
- Assign each patient a number between 1.0 and 5.0 (decimals included) corresponding to how unwell or “sick” you consider them to be, using the following ASA-inspired scale:
    1. Healthy
    2. Mild systemic insult
    3. Severe systemic insult but stable
    4. Unstable patient
    5. Moribund without immediate intervention; peri-arrest
- Rank the patients in the order you would review them
- Maximum allocated time: 20 minutes

Waiting slide

Move to next slide once the task has been completed to the participant’s satisfaction or maximum allocated time has passed

Task B: Questions to participant

1. How did you find the process of assessing and prioritising the patients?

2. How difficult or complex would you say the cases were?

3. Any thoughts about how the clinical information was presented throughout the task?

4. How did you find using the tool during the task?

5. Did you feel you understood what the tool does and what its output represents? Do you think understanding this is important?

6. What did you think about the number scale and granularity of the tool's output? Would you prefer a simpler range, e.g. 1-10?

7. Are there any patients where you think the tool "got it wrong" or misled you?

8. If you had to assess another 10 patients the same way, would you prefer to have the information from the tool there, or not have it at all?

9. Would the tool be useful for you in clinical practice? Would you have any concerns about using it?

Comparison to alternative tool

Questions to participant

1. What is your impression of the alternative tool?
2. What do you think is your preferred way of doing this task - no tool, your original tool, or the alternative tool?
3. Which of the tools would you prefer to use in clinical practice?
4. What would increase your trust in these tools?

End of interview
